# Geographic Variation of Racial and Ethnic Differences in Uterine Cancer Survival

**DOI:** 10.1001/jamanetworkopen.2025.7227

**Published:** 2025-04-25

**Authors:** Caitlin E. Meade, Jennifer A. Sinnott, Tasleem J. Padamsee, Megan A. Clarke, Jesse J. Plascak, Casey M. Cosgrove, Britton Trabert, Ashley S. Felix

**Affiliations:** 1 Division of Epidemiology, College of Public Health, The Ohio State University, Columbus; 2 Department of Statistics, College of Arts and Sciences, The Ohio State University, Columbus; 3 Division of Health Services Management and Policy, College of Public Health, The Ohio State University, Columbus; 4 Division of Cancer Epidemiology and Genetics, National Cancer Institute, Rockville, Maryland; 5 Division of Cancer Prevention and Control, Department of Internal Medicine, The Ohio State University Comprehensive Cancer Center, James Cancer Hospital and Solove Research Institute, Columbus; 6 Department of Obstetrics and Gynecology, Division of Gynecologic Oncology, The Ohio State University Wexner Medical Center, Arthur G James Cancer Center, Columbus; 7 Department of Obstetrics and Gynecology, Spencer Fox Eccles School of Medicine, University of Utah, Salt Lake City; 8 Huntsman Cancer Institute, University of Utah, Salt Lake City

## Abstract

**Importance:**

Racial and ethnic disparities in uterine cancer survival are well-documented; however, limited data exist regarding the interplay of geography, diversity, and race and ethnicity in survival disparities.

**Objective:**

To examine associations of race and ethnicity with uterine cancer–specific survival according to geographic region and regional diversity.

**Design, Setting, and Participants:**

This retrospective cohort study included patients with uterine cancer diagnosed from 2000 to 2019, from 17 Surveillance, Epidemiology, End Results registries, grouped by US location and ranked according to the US Census Bureau’s Diversity Index (DI; range, 0%-100%; higher values indicate greater diversity), a metric of racial and ethnic composition. Analyses were conducted from June 8, 2024 to October 30, 2024.

**Exposures:**

Race and ethnicity of patients with uterine cancer, categorized as Asian, Black, Hispanic, and White.

**Main Outcomes and Measures:**

Cox proportional hazards models were used to estimate hazard ratios (HRs) and 95% CIs for multivariable-adjusted associations of race and ethnicity with uterine cancer–specific survival (primary outcome) in the overall sample and stratified by location. Location-stratified models were used to examine whether associations of race and ethnicity with survival varied by tumor characteristics.

**Results:**

Among 162 500 patients with uterine cancer (median [IQR] age at diagnosis, 61 [54-69] years), there were 12 226 Asian patients (7.5%), 14 007 Black patients (8.6%), 20 799 Hispanic patients (12.8%), and 115 468 White patients (71.1%). Cancer-specific survival was better among Asian patients (HR, 0.91; 95% CI, 0.86-0.97), worse among Black patients (HR, 1.34; 95% CI, 1.28-1.40), and not different among Hispanic patients (HR, 1.01; 95% CI, 0.97-1.06) compared with White patients. Location-stratified analyses found worse uterine cancer–specific survival among Black patients compared with White patients in both higher DI locations (California: HR, 1.34; 95% CI, 1.25-1.44; DI, 69.7%; New Jersey: HR, 1.34; 95% CI, 1.21-1.50; DI, 65.8%; Georgia: HR, 1.39; 95% CI, 1.26-1.53; DI = 64.1%) and lower DI locations (Louisiana: HR, 1.34; 95% CI, 1.16-1.54; DI = 58.6%; Connecticut: HR, 1.42; 95% CI, 1.17-1.72; DI, 55.7%; Iowa: HR, 1.71; 95% CI, 1.01-2.89; DI, 30.8%). Hispanic patients, compared with White patients, had worse survival in Hawaii (HR, 2.09; 95% CI, 1.28-3.42) and Georgia (HR, 1.44; 95% CI, 1.13-1.82), whereas Asian patients had better survival than White patients in California (HR, 0.91; 95% CI, 0.84-0.97). In locations demonstrating survival disparities between Black and White patients, these patterns were evident in most tumor characteristic–defined strata.

**Conclusions and Relevance:**

In this cohort study of patients with uterine cancer, racial and ethnic disparities in survival within specific geographic areas were identified. Targeted research may reduce national disparities.

## Key Points

**Question:**

How do racial and ethnic disparities in uterine cancer survival vary geographically in the US?

**Findings:**

In this cohort study of 162 500 patients with uterine cancer, uterine cancer–specific survival was better among Asian patients, worse among Black patients, and not different among Hispanic patients compared with White patients. Location-stratified analyses comparing Asian, Black, and Hispanic patients with White patients with uterine cancer showed disparate survival within less and more diverse US locations; however, associations varied by race and ethnicity.

**Meaning:**

These findings suggest that race- and ethnicity-based survival differences among patients with uterine cancer varied within the US, providing clues on the settings where future research should be targeted.

## Teaser

This cohort study examines associations of race and ethnicity with uterine cancer survival by geographic region and regional diversity.

## Introduction

Unlike other common cancers in which 5-year relative survival is improving, uterine cancer—the most common gynecologic cancer in the US—is characterized by declining 5-year relative survival.^1^ This observation is associated with increasing incidence of poor prognosis subtypes (ie, nonendometrioid histologies) over the last 2 decades.^2^ In addition to the overall decrease in 5-year relative uterine cancer survival, persistent gaps between racial and ethnic groups exist: 5-year relative survival is worse among Black patients with uterine cancer (63.2%) compared with Asian (83.7%), Hispanic (81.4%), and White (86.1%) patients.^2^ Diagnosis delays,^3^ lack of guideline-concordant treatment,^4^ and more advanced disease and aggressive tumor histology^5^ contribute to, but do not fully explain, the complex interaction of social, structural, clinical, and individual factors that perpetuate uterine cancer survival disparities.

Despite well-documented racial and ethnic disparities in uterine cancer survival at the national level, limited data exist regarding the interplay of geographic region with racial and ethnic disparities in uterine cancer survival. Clarke and colleagues^2^ evaluated 5-year relative survival among Black, Hispanic, and White patients with uterine cancer stratified by broad US region (Northeast, Midwest, South, and West), stage, and histology and reported no regional differences in relative survival among Black and Hispanic patients with uterine cancer compared with their age-, race- and ethnicity-, calendar year–, and county-matched counterparts. More recently, a variable association of county-level socioeconomic status (SES) with 5-year uterine cancer relative survival by region was identified, with stronger associations between worse county-level SES and poor survival noted in the Northeast.^6^ While this analysis did not explore the joint impact of area-level SES and race and ethnicity on geographic distributions of 5-year relative survival, these results suggest that uterine cancer survival is spatially patterned according to county-level characteristics, which are highly correlated with race and ethnicity.^7^

We sought to build on this work and examine geographic variability in associations of race and ethnicity with uterine cancer-specific survival in analyses adjusted for tumor characteristics and treatment, factors known to vary regionally. Moreover, because the racial and ethnic distribution of people in the US is driven by complex factors, we present results according to location diversity. We posit that area-level diversity offers the opportunity to explore impacts of systemic discrimination (ie, discrimination in the form of historical and contemporary policies, structures, and interactional patterns that disadvantage racially and ethnically minoritized groups, including Asian, Black, and Hispanic populations) that may be easier to observe in more diverse areas because of the potential to impact higher numbers of people. As such, we hypothesized that the magnitude of racial and ethnic disparities in survival would be larger in US locations with greater racial and ethnic diversity, owing to the larger potential for economic and social gaps between minority and majority populations to manifest in poor health outcomes. We also explored whether geographically stratified associations of race and ethnicity with cancer-specific survival were modified by histology and stage at diagnosis. In line with the notion that variability in treatment access results in greater disparities in conditions where treatment has the highest impact (ie, higher treatment amenability),^8^ we hypothesized that racial and ethnic survival disparities would be largest for less aggressive subtypes (eg, low-grade endometrioid and early stage disease) that are highly amenable to treatment.

## Methods

This cohort study was considered not human participants research by the Ohio State University institutional review board and thus was exempt from informed consent. We followed the Strengthening the Reporting of Observational Studies in Epidemiology (STROBE) reporting guideline.

### Data Source

We used the Surveillance, Epidemiology, and End Results (SEER) Research Plus Data, 17 Registries (SEER 17), excluding the Alaska Native Tumor Registry due to the absence of Asian, Black, Hispanic, and White patients. Data from these registries cover approximately 26.5% of the US population.^9^ We used SEER 17 instead of SEER 22 due to unavailable patient-level survival data in the latter.

### Participants

A retrospective cohort study was conducted of adult (age ≥18 years) females diagnosed with first primary uterine cancer (*International Classification of Disease for Oncology, Third Edition* [*ICD-O-3*] primary site codes C54.0-C54.9, C55.9) between 2000 and 2019 resulting in an initial sample of 201 679 patients. We excluded patients from the Alaska Native Tumor Registry (142 patients) and restricted to the following racial and ethnic groups: Hispanic, non-Hispanic Asian, non-Hispanic Black, and non-Hispanic White (4812 patients excluded). Histology types included endometrioid, mucinous, or adenocarcinoma (*ICD-O-3* morphology codes 8140, 8380-8383, 8210, 8211, 8261-8263, 8480-8482, 8560, 8570); serous (*ICD-O-3* codes 8050, 8260, 8441, 8460, 8461); carcinosarcoma (*ICD-O-3* codes 8950, 8951, 8980, 8981), mixed epithelial (*ICD-O-3* codes 8323, 8255); clear cell (*ICD-O-3* codes 8310); or sarcoma (*ICD-O-3* codes 8800-8806, 8810, 8811, 8814, 8815, 8890, 8891, 8895-8897, 8900-8902, 8910, 8920, 8930, 8931, 8933-8935) (6364 patients). Further exclusions included unknown or noninvasive stage (16 843 patients), no or unknown surgery (9706 patients), unknown cause of death (734 patients), and unknown survival time (578 patients). After exclusions, 162 500 patients remained in the analysis.

### Primary Outcome

The primary study outcome was uterine cancer–specific survival. Uterine cancer–specific survival was calculated using the SEER cause of death to site recode variable (values of *corpus uteri* and *uterus not otherwise specified*), vital status, and survival months.

### Exposure

Ethnicity was determined by the North American Association of Central Cancer Registries Hispanic Identification Algorithm that uses a combination of variables including maiden name, surname, birthplace, and medical records,^10^ and race was self-reported. We cross-classified race categories of Asian (including Asian Indian, Asian Indian or Pakistani not otherwise specified [NOS]; Chinese, Japanese, Filipino, Hmong, Kampuchean [including Khmer and Cambodian], Korean, Laotian, Pakistani, Thai, Vietnamese, and other Asian [including Asian NOS]), Black, and White and ethnicities of Hispanic and non-Hispanic to produce the categories Hispanic ethnicity of any race, non-Hispanic Asian (hereafter, *Asian*), non-Hispanic Black (hereafter, *Black*), and non-Hispanic White (hereafter, *White*).

### Covariates

The 16 SEER registries were grouped according to state (California, Connecticut, Georgia, Hawaii, Iowa, Kentucky, Louisiana, New Jersey, New Mexico, Utah) or city (Seattle-Puget Sound). Additional covariates included age at diagnosis (<50, 50-69, ≥70 years; or continuous), median county-level income (<$75 000, ≥$75 000), marital status (married, unmarried), year of diagnosis (2000-2004, 2005-2009, 2010-2014, 2015-2019), surgery (tumor destruction or excision, subtotal or supracervical hysterectomy, total abdominal hysterectomy with or without bilateral salpingo-oophorectomy, modified radical or extended hysterectomy, surgery NOS), stage (I, II, III, IV), chemotherapy (yes, no or unknown), and radiation (none, external beam radiotherapy [EBRT], vaginal brachytherapy [VBT], combination). Grade and histology were combined and categorized as low-grade endometrioid, high-grade endometrioid, unknown grade endometrioid, serous, carcinosarcoma, clear cell, mixed epithelial, and sarcoma. Variables with missing data were included as a separate unknown category. SEER-coded rural/urban location was categorized as urban (large metropolitan county [population >1 million], medium metropolitan county [population 250 000-1 million], small metropolitan county [population <250 000]) or rural (nonmetropolitan counties adjacent to a metropolitan area, nonmetropolitan counties not adjacent to a metropolitan area).

### Statistical Analysis

Demographic, tumor, and treatment characteristics were summarized according to race and ethnicity and US area separately. Kaplan-Meier curves and log-rank tests were used to compare survival distributions according to race and ethnicity in the overall sample and by US location. Cox proportional hazards models were used to estimate hazard ratios (HRs) and 95% CIs for multivariable-adjusted associations between race and ethnicity and cancer-specific survival in the overall sample and stratified by location. We displayed heterogeneity in race and ethnicity survival associations using forest plots with locations ranked according to the 2020 US Census Bureau’s Diversity Index (DI).^11^ The DI ranks areas according to the probability that 2 randomly selected people within the population are from different race and ethnicity groups. The DI ranges from 0% to 100% with a value of 0% indicating that everyone in the population is the same race and ethnicity and values closer to 100% indicating that everyone in the population are racially and ethnically different. In this sample, Hawaii was the most diverse (DI score, 76.0%) and Iowa was the least diverse (DI score, 30.8%). We considered the 6 locations with the highest DI scores as more diverse and the 5 locations with the lowest DI scores as less diverse.

In exploratory analyses, we present location-specific Cox regression models further stratified by histology (low-grade endometrioid, high-grade endometrioid, nonendometrioid [serous, carcinosarcoma, clear cell, mixed epithelial], and sarcoma) or stage (early stage [I] and advanced stage [II, III, IV]). All models were adjusted for the following factors selected a priori: categorical age at diagnosis, median county-level income, marital status, metropolitan status, year of diagnosis, histology, stage, surgery, chemotherapy, and radiation. In the respective histology- and stage-stratified models, histology and stage were not included as adjustment factors. Proportional hazards assumptions were examined visually with plots of the scaled Schoenfeld residuals vs log follow-up time for the main exposure (ie, race and ethnicity), and we observed no violations. Statistical analyses were performed using SAS version 9.4 (SAS Institute). All *P* values were 2-sided; statistical significance was set at *P* < .05. Because of the descriptive and exploratory nature of this analysis, we did not adjust for multiple comparisons. For perspective, 36 comparisons were made to test our primary hypotheses of racial and ethnic survival disparities in location-stratified analyses (eFigure in Supplement 1). Data were analyzed from June 8, 2024 to October 30, 2024.

## Results

Among 162 500 patients with uterine cancer, median (IQR) age at diagnosis was 61 (54-69) years. The analysis included 12 226 Asian patients (7.5%), 14 007 Black patients (8.6%), 20 799 Hispanic patients (12.8%), and 115 468 White patients (71.1%). Asian, Black, and Hispanic patients more commonly lived in urban areas than White patients. Asian patients were more likely to live in higher income areas (8730 patients [71.4%]), compared with Black (5585 patients [39.9%]), Hispanic (10 078 patients [48.5%]), and White (65 573 patients [56.8%]) patients. Additionally, Black patients, compared with White patients, were more likely to be diagnosed with aggressive histology types, such as serous (2225 patients [15.9%] vs 6323 patients [5.5%]), carcinosarcoma (1340 patients [9.6%] vs 3161 patients [2.7%]), and sarcoma (708 patients [5.1%] vs 1961 patients [1.7%]), and advanced-stage tumors (stage III: 2248 patients [16.1%] vs 13 048 patients [11.3%]; stage IV: 1537 patients [11.0%] vs 5874 patients [5.1%]) but were less likely to receive guideline-concordant surgery (total abdominal hysterectomy with or without bilateral salpingo-oophorectomy: 12 637 patients [90.2%] vs 107 664 patients [93.2%]). Asian patients were more likely to reside in California (8484 patients [69.4%] vs 42 012 patients [36.4%]) or Hawaii (1575 patients [12.9%] vs 714 patients [0.6%]) than White patients, and Hispanic patients were more likely to reside in California (15 149 patients [72.8%] vs 42 012 patients [36.4%]) or New Mexico (1248 patients [6.0%] vs 2175 patients [1.9%]) than White patients. Black patients were more likely to reside in southern states, including Georgia (4326 patients [30.9%] vs 10 848 patients [9.4%]) and Louisiana (1961 patients [14.0%] vs 4669 patients [4.0%]) compared with White patients (Table). The eTable in Supplement 1 presents characteristics of patients with uterine cancer according to the 11 included locations.

**Table.** Characteristics of Patients With Uterine Cancer According to Race and Ethnicity, 2000-2019

Characteristic	Patients, No. (%)			
	Asian (n = 12 226)	Black (n = 14 007)	Hispanic (n = 20 799)	White (n = 115 468)
Geographic region				
Hawaii	1575 (12.9)	15 (0.1)	150 (0.7)	714 (0.6)
California	8484 (69.4)	3912 (27.9)	15 149 (72.8)	42 012 (36.4)
New Jersey	931 (7.6)	2197 (15.7)	2024 (9.7)	17 841 (15.5)
Georgia	270 (2.2)	4326 (30.9)	571 (2.8)	10 848 (9.4)
New Mexico	38 (0.3)	51 (0.4)	1248 (6.0)	2175 (1.9)
Seattle (Puget Sound), Washington	597 (4.9)	256 (1.8)	349 (1.7)	8582 (7.4)
Louisiana	67 (0.6)	1961 (14.0)	146 (0.7)	4669 (4.0)
Connecticut	125 (1.0)	683 (4.9)	689 (3.3)	8011 (6.9)
Utah	57 (0.5)	19 (0.1)	290 (1.4)	3864 (3.4)
Kentucky	47 (0.4)	495 (3.5)	81 (0.4)	8775 (7.6)
Iowa	35 (0.3)	92 (0.7)	102 (0.5)	7977 (6.9)
Age, y				
<50	2682 (21.9)	1713 (12.2)	5319 (25.6)	12 238 (10.6)
50-69	7697 (63.0)	9060 (64.7)	12 281 (59.1)	72 481 (62.8)
≥70	1847 (15.1)	3234 (23.1)	3199 (15.4)	30 749 (26.6)
Age, median (IQR), y	57 (51-65)	62 (56-69)	58 (49-65)	62 (56-70)
Area-level annual income, $				
<75 000	3496 (28.6)	8422 (60.1)	10 720 (51.5)	49 888 (43.2)
≥75 000	8730 (71.4)	5585 (39.9)	10 078 (48.5)	65 573 (56.8)
Unknown	0	0	1 (<0.1)	7 (<0.1)
Marital status				
Unmarried	4106 (33.6)	8793 (62.8)	9464 (45.5)	47 826 (41.4)
Married	7643 (62.5)	4445 (31.7)	10 452 (50.3)	62 850 (54.4)
Unknown	477 (3.9)	769 (5.5)	883 (4.3)	4792 (4.2)
Rural/urban location				
Urban	11 899 (97.3)	12 790 (91.3)	20 087 (96.6)	99 293 (86.0)
Rural	327 (2.7)	1217 (8.7)	711 (3.4)	16 168 (14.0)
Unknown	0	0 (0.0)	1 (<0.1)	7 (<0.1)
Year of diagnosis				
2000-2004	1718 (14.1)	1798 (12.8)	2623 (12.6)	22 812 (19.8)
2005-2009	2379 (19.5)	2551 (18.2)	3807 (18.3)	26 679 (23.1)
2010-2014	3543 (29.0)	4236 (30.2)	6076 (29.2)	32 229 (27.9)
2015-2019	4586 (37.5)	5422 (38.7)	8293 (39.9)	33 748 (29.2)
Histology				
Low-grade endometrioid	7157 (58.5)	5540 (39.6)	12 312 (59.2)	70 750 (61.3)
High-grade endometrioid	1254 (10.3)	1691 (12.1)	1889 (9.1)	11 546 (10.0)
Unknown grade endometrioid	1494 (12.2)	1356 (9.7)	2444 (11.8)	14 433 (12.5)
Serous	770 (6.3)	2225 (15.9)	1340 (6.4)	6323 (5.5)
Carcinosarcoma	372 (3.0)	1340 (9.6)	719 (3.5)	3161 (2.7)
Clear cell	179 (1.5)	308 (2.2)	269 (1.3)	1238 (1.1)
Mixed epithelial	668 (5.5)	839 (6.0)	1058 (5.1)	6056 (5.2)
Sarcoma	332 (2.7)	708 (5.1)	768 (3.7)	1961 (1.7)
Tumor stage				
I	9140 (74.8)	9123 (65.1)	15 708 (75.5)	89 869 (77.8)
II	717 (5.9)	1099 (7.9)	1336 (6.4)	6677 (5.8)
III	1599 (13.1)	2248 (16.1)	2471 (11.9)	13 048 (11.3)
IV	770 (6.3)	1537 (11.0)	1284 (6.2)	5874 (5.1)
Surgery				
Tumor destruction or excision	108 (0.9)	179 (1.3)	225 (1.1)	701 (0.6)
Subtotal or supracervical hysterectomy	141 (1.2)	211 (1.5)	343 (1.7)	1250 (1.1)
Total abdominal hysterectomy with or without bilateral salpingo-oophorectomy	11 252 (92.0)	12 637 (90.2)	19 031 (91.5)	107 664 (93.2)
Modified radical or extended hysterectomy	710 (5.8)	944 (6.7)	1167 (5.6)	5678 (4.9)
Surgery, not otherwise specified	15 (0.1)	36 (0.3)	33 (0.2)	175 (0.2)
Chemotherapy				
No or unknown	9734 (79.6)	9646 (68.9)	16 892 (81.2)	96 582 (83.6)
Yes	2492 (20.4)	4361 (31.1)	3907 (18.8)	18 886 (16.4)
Radiation				
None	9162 (74.9)	9447 (67.4)	15 457 (74.3)	83 081 (72.0)
External beam radiation therapy	1307 (10.7)	1696 (12.1)	2235 (10.8)	11 329 (9.8)
Vaginal brachytherapy	1029 (8.4)	1671 (11.9)	1795 (8.6)	13 654 (11.8)
External beam radiation therapy with vaginal brachytherapy	581 (4.8)	891 (6.4)	948 (4.6)	5897 (5.1)
Unknown	147 (1.2)	302 (2.2)	364 (1.8)	1507 (1.3)

**Figure 1.**
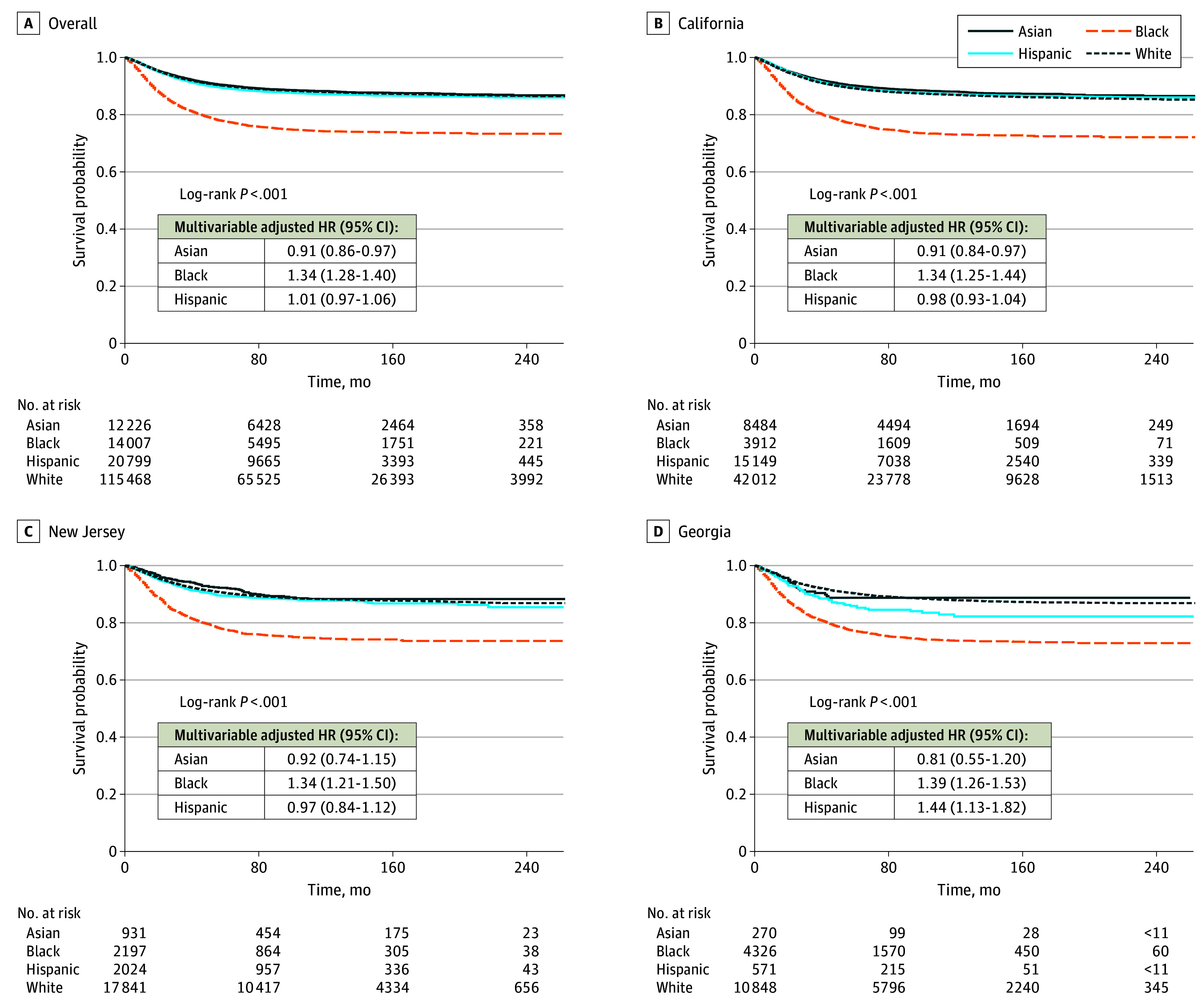
Kaplan-Meier Survival Curves Race and Ethnicity and Uterine Cancer–Specific Survival in the Overall Sample, California, New Jersey, and Georgia Compared with White patients, Asian patients showed improved or similar survival; Black patients experienced worse uterine survival in the overall sample, California, New Jersey, and Georgia; and Hispanic patients experienced worse survival in Georgia. HR indicates hazard ratio.

Median (IQR) follow-up was 84 (42-144) months for Asian patients, 59 (28-114) months for Black patients, 73 (35-130) months for Hispanic patients, and 93 (47-153) months for White patients. Figure 1 shows Kaplan-Meier survival curves and multivariable-adjusted HRs for associations of race and ethnicity with cancer-specific survival in the overall sample, California, New Jersey, and Georgia, while the eFigure in Supplement 1 provides estimates overall and for each location, with results presented in order from highest DI score (ie, most diverse) to lowest DI score (ie, least diverse). Compared with White patients in the overall sample, cancer-specific survival was better among Asian patients (HR, 0.91; 95% CI, 0.86-0.97), worse among Black patients (HR, 1.34; 95% CI, 1.28-1.40), and not different among Hispanic patients (HR, 1.01; 95% CI, 0.97-1.06). Variation in racial and ethnic survival disparities according to location was evident; however, clear patterns according to DI score were not identified. For example, Black patients had worse cancer-specific survival than White patients in California (HR, 1.34; 95% CI, 1.25-1.44), New Jersey (HR, 1.34; 95% CI, 1.21-1.50), and Georgia (HR, 1.39; 95% CI, 1.26-1.53), ie, locations with higher DI scores, and worse survival in Louisiana (HR, 1.34; 95% CI, 1.16-1.54), Connecticut (HR, 1.42; 95% CI, 1.17-1.72), and Iowa (HR, 1.71; 95% CI, 1.01-2.89), ie, locations with lower DI scores. Compared with White patients, Hispanic patients had worse survival in Hawaii (HR, 2.09; 95% CI, 1.28-3.42) and Georgia (HR, 1.44; 95% CI, 1.13-1.82), whereas Asian patients had better survival in California (HR, 0.91; 95% CI, 0.84-0.97).

**Figure 2.**
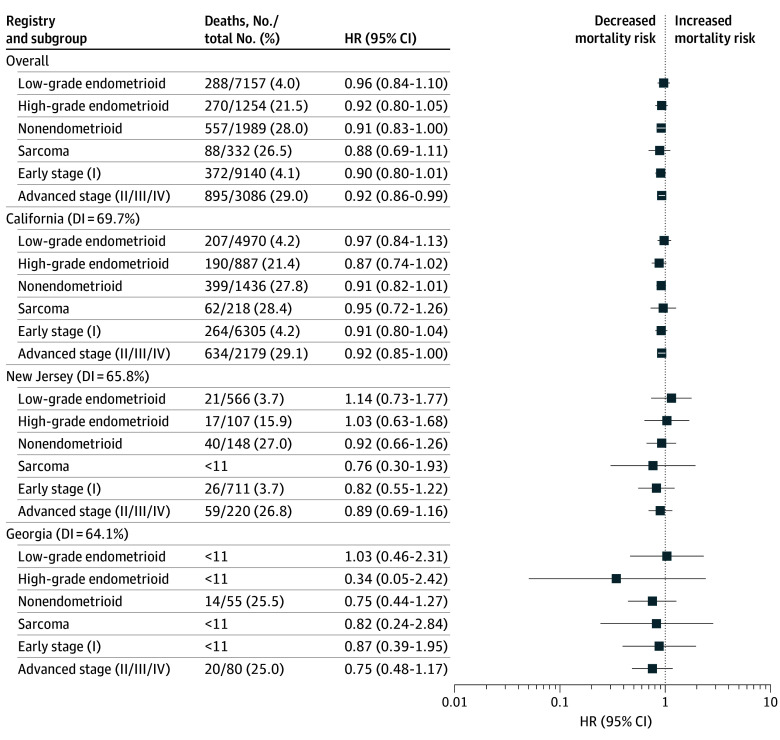
Association of Race and Ethnicity With Uterine Cancer–Specific Survival Among Asian Patients Stratified by Histology and Stage in the Overall Sample, California, New Jersey, and Georgia White patients are the reference group for all comparisons. Asian patients diagnosed with nonendometrioid or advanced-stage tumors had better cancer-specific survival. Within the California registry, Asian patients diagnosed with advanced-stage tumors had better cancer-specific survival. Diversity index (DI) ranged from 0% to 100%, with higher percentage indicating more diversity. HR indicates hazard ratio.

**Figure 3.**
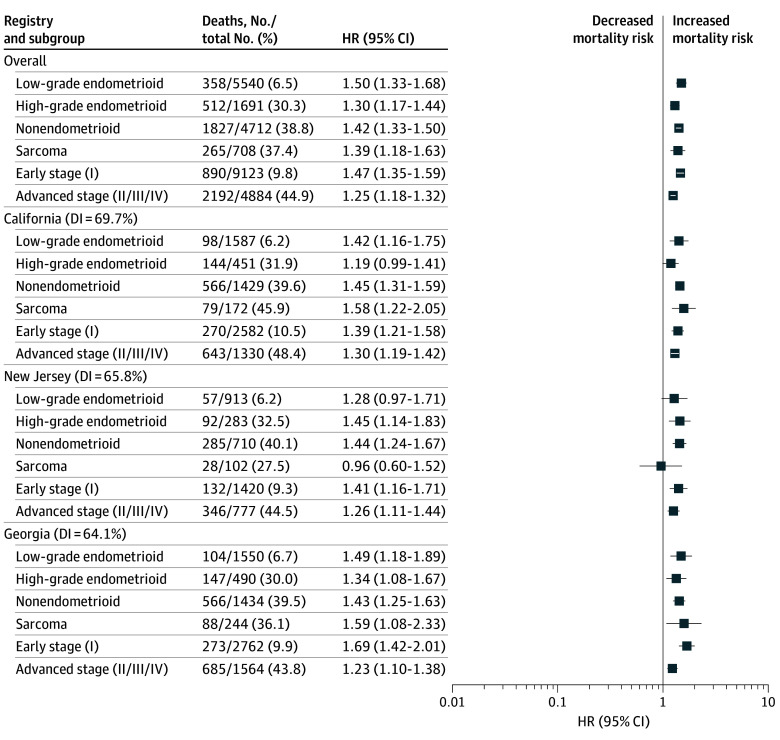
Association of Race and Ethnicity With Survival Among Black Patients Stratified by Histology and Stage in the Overall Sample, California, New Jersey, and Georgia White patients are the reference group for all comparisons. Black patients had worse uterine cancer–specific survival in all histology and stage subgroups. Worse cancer-specific survival was also noted for Black patients in 1 or more tumor-defined subgroups within Georgia, California, and New Jersey. Diversity index (DI) ranged from 0% to 100%, with higher percentage indicating more diversity. HR indicates hazard ratio.

**Figure 4.**
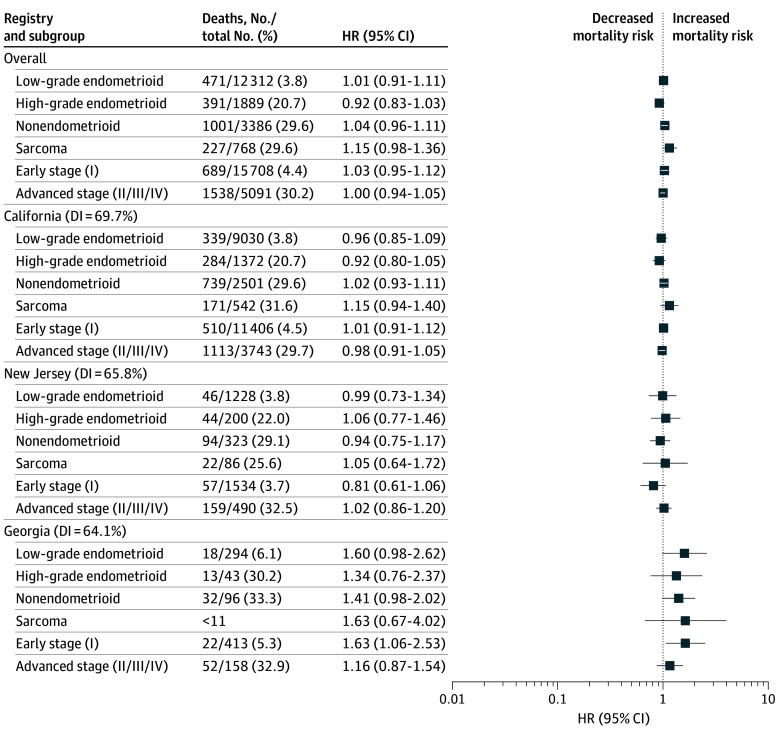
Association of Race and Ethnicity With Survival Among Hispanic Patients Stratified by Histology and Stage in the Overall Sample, California, New Jersey, and Georgia White patients are the reference group for all comparisons. No significant differences were noted among Hispanic patients in the overall sample. Within the Georgia registry, Hispanic patients diagnosed with early-stage tumors had worse cancer-specific survival. Diversity index (DI) ranged from 0% to 100%, with higher percentage indicating more diversity. HR indicates hazard ratio.

Figure 2, Figure 3, and Figure 4, along with eFigures 2 through 4 in Supplement 1, present HRs for cancer-specific survival associations comparing each racial and ethnic group with White patients as the reference group, in subgroups defined by histology (eg, low-grade endometrioid) or stage (eg, early) in the overall sample and stratified by location. Few differences were observed when comparing Asian and White patients in the overall sample (Figure 2; eFigure 2 in Supplement 1). Compared with White patients, Asian patients diagnosed with nonendometrioid (HR, 0.91; 95% CI, 0.83-1.00) or advanced-stage (HR, 0.92; 95% CI, 0.86-0.99) tumors had better cancer-specific survival. Within the California registry, Asian patients diagnosed with advanced-stage tumors had better cancer-specific survival than White patients (HR, 0.92; 95% CI, 0.85-1.00).

Comparisons demonstrated worse cancer-specific survival among Black patients than White patients in all histology and stage subgroups in the overall sample (Figure 3; eFigure 3 in Supplement 1); however, estimates were highest for patients with low-grade endometrioid (HR, 1.50; 95% CI, 1.33-1.68) and early-stage (HR, 1.47; 95% CI, 1.35-1.59) tumors. In Georgia, California, New Jersey, and Connecticut, worse survival for Black patients compared with White patients was observed in most tumor-defined subgroups. In Louisiana, worse cancer-specific survival was noted for Black patients compared with White patients with low-grade endometrioid (HR, 2.08; 95% CI, 1.54-2.82), nonendometrioid (HR, 1.29; 95% CI, 1.04-1.60), and early-stage (HR, 1.77; 95% CI, 1.38-2.28) disease. Worse cancer-specific survival was observed for Black patients compared with White patients with high-grade endometrioid disease in Seattle (HR, 2.23; 95% CI, 1.03-4.83) and nonendometroid disease in Iowa (HR, 2.01; 95% CI, 1.00-4.01).

In the comparison of Hispanic patients with White patients, no significant differences were noted in the overall sample (Figure 4; eFigure 4 in Supplement 1). Location-stratified results showed that Hispanic patients with nonendometroid disease had worse cancer-specific survival in Hawaii (HR, 3.99; 95% CI, 1.72-9.25) and Kentucky (HR, 3.82; 95% CI, 1.53-9.57), while those diagnosed with high-grade endometrioid tumors had worse cancer-specific survival in Iowa (HR, 5.53; 95% CI, 1.27-24.03). Among patients with sarcoma, worse cancer-specific survival was observed for Hispanic patients than White patients in Louisiana (HR, 4.52; 95% CI, 1.32-15.44) and Iowa (HR, 4.72; 95% CI, 1.30-17.08). Hispanic patients with early-stage tumors had worse cancer-specific survival in Georgia (HR, 1.63; 95% CI, 1.06-2.53) while those with advanced-stage tumors had worse cancer-specific survival in Hawaii (HR, 3.28; 95% CI, 1.75-6.13) compared with White patients.

## Discussion

In this large, population-based cohort study of patients with uterine cancer, we found place-based heterogeneity in associations of race and ethnicity with cancer-specific survival. In line with national survival estimates, our unstratified analyses showed that compared with White patients with uterine cancer, survival was better among Asian patients, worse among Black patients, and not different among Hispanic patients.^2,12^ Although differences according to geography were present, no clear patterns according to location diversity were identified. For example, survival disparities between Black and White patients were observed in more diverse (eg, California) and less diverse locations (eg, Connecticut). Within these locations, survival disparities between Black and White patients were evident within almost all tumor characteristic–defined strata, including those with low-grade endometrioid or early-stage disease. Together, our results indicate that Black patients with uterine cancer had worse outcomes regardless of the diversity index, pointing to structural inequities at a regional or national level that may not be influenced by diversity within a state. Relative to comparisons between Black and White patients, we observed fewer survival disparities when comparing Asian and Hispanic patients with White patients. Describing geographical variation is important in highlighting specific areas requiring further etiologic research and targeted interventions.

Limited population-based research exists on racial and ethnic differences in uterine cancer–specific survival according to geographic region. In the US, distributions of people according to race and ethnicity vary significantly by geography owing to many factors, including settlement patterns, immigration and internal migration patterns, and labor opportunities. Moreover, area-level factors, such as poverty, education, and access to care, vary spatially and are associated with race and ethnicity^7^ and cancer survival.^13^ Our hypothesis that uterine cancer survival disparities would be worse in areas with greater diversity reflects the notion that racially and ethnically minoritized people experience disadvantages in numerous domains that also impact health outcomes. With the available data, we were unable to specifically assess whether concentrated disadvantages in macro-level conditions (eg, intergenerational wealth, home ownership, educational status, occupation, health literacy, experiences of discrimination) mediate racial and ethnic disparities in uterine cancer survival. In other health-related analyses examining geographic variations in racial and ethnic disparities, greater disparities in prostate cancer survival,^14^ stroke thrombolysis,^15^ and influenza mortality^16^ were identified in locations with larger proportions of racially and ethnically minoritized people, potentially reflecting the impact of structural, historical, and contemporary racism as a ubiquitous mechanism impacting multiple health outcomes.^17,18^

Additionally, our hypothesis that disparities would be largest in tumor subgroups with higher treatment amenability was somewhat substantiated for Black patients. In the overall study population, worse survival was noted for Black patients compared with White patients within all tumor-defined subgroups; however, survival disparities were largest for patients with low-grade endometrioid or early-stage disease. Surgical intervention is the mainstay of treatment for patients with these tumor features, resulting in a 5-year relative survival rate of 90%.^2^ Enduring treatment disparities have been noted in the uterine cancer population,^4,19^ and our results highlight specific areas in which future etiologic work might be directed. As a hypothetical example, if survival disparities between Black and White patients with low-grade endometrioid disease residing in Georgia were mediated by lower use of minimally invasive hysterectomy procedures among Black patients, research on the etiology of this treatment disparity and efforts to safely increase its use among Black patients would be warranted.

Attempts at understanding racial and ethnic heterogeneity in uterine cancer survival have focused on differences in tumor biology, comorbidities, and treatment receipt within Black and White patients. Our descriptive analysis, while unable to provide mechanisms for the observed geographically specific survival disparities, suggests that uterine cancer disparities may be setting-specific and more pronounced among patients with certain tumor characteristics. This observation more strongly implicates aspects of the lived environment that cause systematic disparities, as opposed to genetic differences between groups of people living in various areas of the US.

### Limitations and Strengths

This study has some limitations. In addition to the descriptive nature of this work, other limitations include the limited focus on the 11 locations with SEER registries. Additionally, it is possible the lack of racial and ethnic disparities in uterine cancer–specific survival within some areas, specifically those with a lower DI score, was due to low power consequent to lower proportions of minoritized racial and ethnic populations in these areas. The absence of other measures of structural inequities and systemic discrimination to contextualize our location-specific findings is a limitation. As discussed by Hing et al,^20^ the interaction of diversity and dissimilarity measures with other domains (eg, housing quality, education) allows for a better understanding of the mechanisms through which area-level diversity may shape health. We also acknowledge that our analyses are limited by decisions to aggregate multiple racial and ethnic groups into broad categories (eg, Asian), which could mask different lived experiences, and to exclude American Indian and Alaska Native patients. These decisions were influenced by low sample sizes.

Despite these limitations, several important strengths warrant mention. First, we used a large, inclusive cancer database, which allowed us to include members of minoritized racial and ethnic groups that are often excluded due to low sample sizes. Furthermore, our analytic approach allowed us to observe granular racial and ethnic survival disparities that would have gone undetected in unstratified models and permitted investigation of hypotheses that disparities may be largest among tumors most amenable to treatment, informing directions for future research and interventions.

## Conclusions

In this cohort study of patients with uterine cancer, racially and ethnically disparate uterine cancer–specific survival was observed in specific geographic locations. While etiologic studies that assess the causes of geographically defined racially and ethnically disparate uterine cancer survival are needed, our findings suggest that locations with the most pronounced racial and ethnic disparities should be prioritized.

## Notes

Supplement 1. **eFigure 1. **Kaplan Meier Survival Curves According to Race and Ethnicity Overall and Stratified by SEER Registry **eTable.** Characteristics of Patients With Uterine Cancer According to SEER Registry, 2000-2019 **eFigure 2.** Association Between Race and Ethnicity and Survival Among Asian Patients Stratified by SEER Registry, Histology, and Stage **eFigure 3.** Association Between Race and Ethnicity and Survival Among Black Patients Stratified by SEER Registry, Histology, and Stage **eFigure 4.** Association Between Race and Ethnicity and Survival Among Hispanic Patients Stratified by SEER Registry, Histology, and Stage

Supplement 2.
Data Sharing Statement
