# Supplementary material for: Geographic Variation of Racial and Ethnic Differences in Uterine Cancer Survival
Source: JAMA Netw Open. 2025 Apr 25;8(4):e257227. doi: 10.1001/jamanetworkopen.2025.7227 (PMC12032568; doi:10.1001/jamanetworkopen.2025.7227)
Supplement: Supplement 1. — eFigure 1. Kaplan Meier Survival Curves According to Race and Ethnicity Overall and Stratified by SEER Registry eTable. Characteristics of Patients With Uterine Cancer According to SEER Registry, 2000-2019 eFigure 2. Association Between Race and Ethnicity and Survival Among Asian Patients Stratified by SEER Registry, Histology, and Stage eFigure 3. Association Between Race and Ethnicity and Survival Among Black Patients Stratified by SEER Registry, Histology, and Stage eFigure 4. Association Between Race and Ethnicity and Survival Among Hispanic Patients Stratified by SEER Registry, Histology, and Stage [file jamanetwopen-e257227-s001.pdf]

Supplemental Online Content

Meade CE, Sinnott JA, Padamsee TJ, et al. Geographic variation of racial and ethnic differences in uterine cancer survival. *JAMA Netw Open*. 2025;8(4):e257227. doi:10.1001/jamanetworkopen.2025.7227

eFigure 1. Kaplan Meier Survival Curves According to Race and Ethnicity Overall and Stratified by SEER Registry

eTable. Characteristics of Patients With Uterine Cancer According to SEER Registry, 2000-2019

eFigure 2. Association Between Race and Ethnicity and Survival Among Asian Patients Stratified by SEER Registry, Histology, and Stage

eFigure 3. Association Between Race and Ethnicity and Survival Among Black Patients Stratified by SEER Registry, Histology, and Stage

eFigure 4. Association Between Race and Ethnicity and Survival Among Hispanic Patients Stratified by SEER Registry, Histology, and Stage

This supplemental material has been provided by the authors to give readers additional information about their work.

© 2025 Meade CE et al. *JAMA Network Open*.

eFigure 1. Kaplan Meier survival curves according to race and ethnicity overall and stratified by SEER registry

eFigure 1 legend. Compared with White patients, Asian patients showed improved or similar uterine cancer-specific survival in the overall sample and California; Black patients experienced worse uterine cancer-specific survival in the overall sample, California, New Jersey, Georgia, Louisiana, Connecticut, and Iowa; and Hispanic patients experienced worse uterine cancer-specific survival in Hawaii and Georgia.

© 2025 Meade CE et al. *JAMA Network Open*.

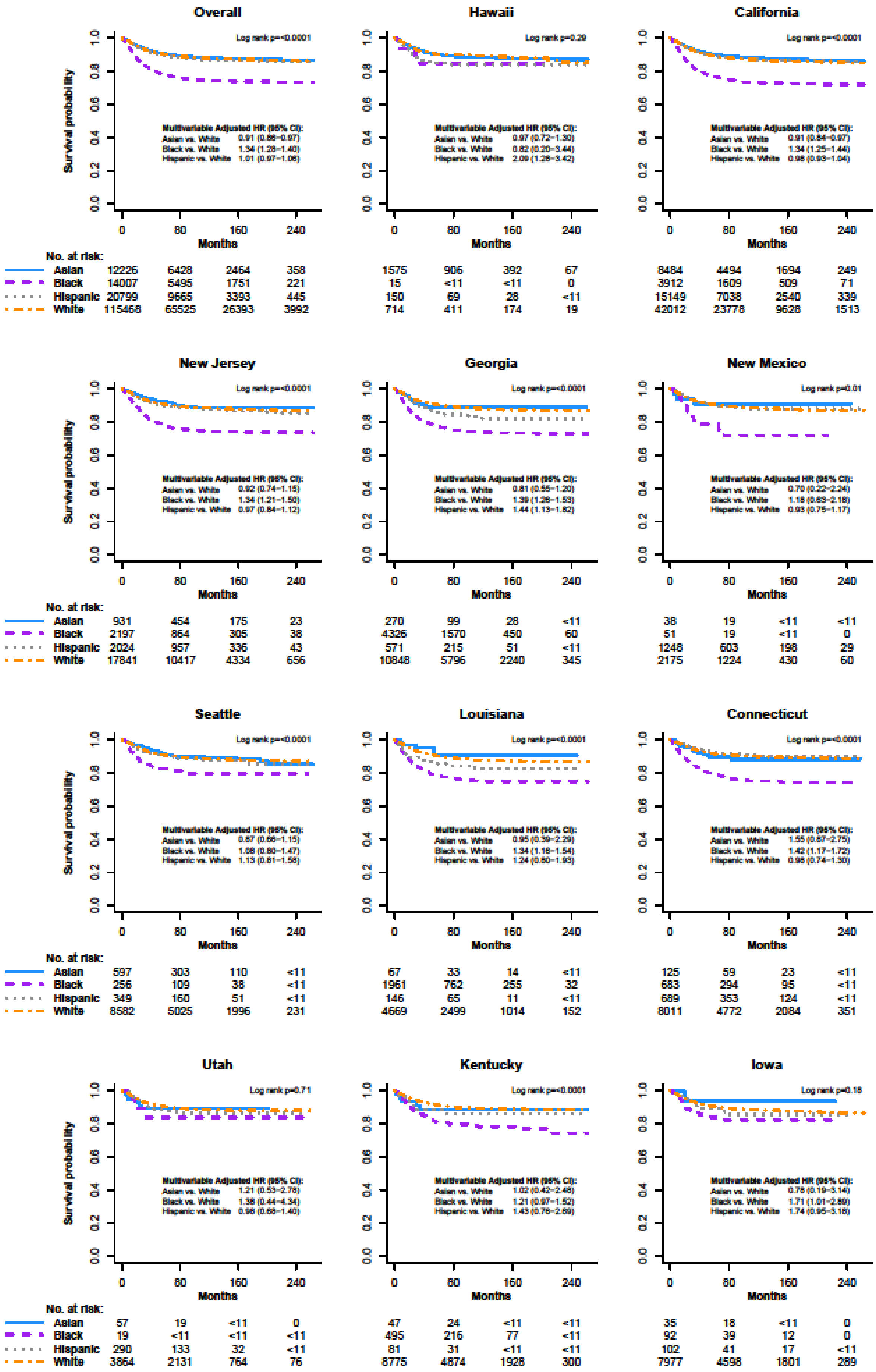
Overall
Log rank p=<0.0001
Multivariable Adjusted HR (95% CI):
Asian vs. White 0.91 (0.86–0.97)
Black vs. White 1.34 (1.28–1.40)
Hispanic vs. White 1.01 (0.97–1.06)
Hawaii
Log rank p=0.29
Multivariable Adjusted HR (95% CI):
Asian vs. White 0.97 (0.72–1.30)
Black vs. White 0.82 (0.20–3.44)
Hispanic vs. White 2.09 (1.28–3.42)
California
Log rank p=<0.0001
Multivariable Adjusted HR (95% CI):
Asian vs. White 0.91 (0.84–0.97)
Black vs. White 1.34 (1.25–1.44)
Hispanic vs. White 0.98 (0.93–1.04)
Survival probability
Months
No. at risk:
Asian 12226 6428 2464 358 | 1575 906 392 67 | 8484 4494 1694 249
Black 14007 5495 1751 221 | 15 <11 <11 0 | 3912 1609 509 71
Hispanic 20799 9665 3393 445 | 150 69 28 <11 | 15149 7038 2540 339
White 115468 65525 26393 3992 | 714 411 174 19 | 42012 23778 9628 1513
New Jersey
Log rank p=<0.0001
Multivariable Adjusted HR (95% CI):
Asian vs. White 0.92 (0.74–1.15)
Black vs. White 1.34 (1.21–1.50)
Hispanic vs. White 0.97 (0.84–1.12)
Georgia
Log rank p=<0.0001
Multivariable Adjusted HR (95% CI):
Asian vs. White 0.81 (0.55–1.20)
Black vs. White 1.39 (1.26–1.53)
Hispanic vs. White 1.44 (1.13–1.82)
New Mexico
Log rank p=0.01
Multivariable Adjusted HR (95% CI):
Asian vs. White 0.70 (0.22–2.24)
Black vs. White 1.18 (0.63–2.18)
Hispanic vs. White 0.93 (0.75–1.17)
No. at risk:
Asian 931 454 175 23 | 270 99 28 <11 | 38 19 <11 <11
Black 2197 864 305 38 | 4326 1570 450 60 | 51 19 <11 0
Hispanic 2024 957 336 43 | 571 215 51 <11 | 1248 603 198 29
White 17841 10417 4334 656 | 10848 5796 2240 345 | 2175 1224 430 60
Seattle
Log rank p=<0.0001
Multivariable Adjusted HR (95% CI):
Asian vs. White 0.87 (0.66–1.15)
Black vs. White 1.08 (0.80–1.47)
Hispanic vs. White 1.13 (0.81–1.58)
Louisiana
Log rank p=<0.0001
Multivariable Adjusted HR (95% CI):
Asian vs. White 0.95 (0.39–2.29)
Black vs. White 1.34 (1.16–1.54)
Hispanic vs. White 1.24 (0.80–1.93)
Connecticut
Log rank p=<0.0001
Multivariable Adjusted HR (95% CI):
Asian vs. White 1.55 (0.87–2.75)
Black vs. White 1.42 (1.17–1.72)
Hispanic vs. White 0.98 (0.74–1.30)
No. at risk:
Asian 597 303 110 <11 | 67 33 14 <11 | 125 59 23 <11
Black 256 109 38 <11 | 1961 762 255 32 | 683 294 95 <11
Hispanic 349 160 51 <11 | 146 65 11 <11 | 689 353 124 <11
White 8582 5025 1996 231 | 4669 2499 1014 152 | 8011 4772 2084 351
Utah
Log rank p=0.71
Multivariable Adjusted HR (95% CI):
Asian vs. White 1.21 (0.53–2.78)
Black vs. White 1.38 (0.44–4.34)
Hispanic vs. White 0.98 (0.68–1.40)
Kentucky
Log rank p=<0.0001
Multivariable Adjusted HR (95% CI):
Asian vs. White 1.02 (0.42–2.48)
Black vs. White 1.21 (0.97–1.52)
Hispanic vs. White 1.43 (0.76–2.69)
Iowa
Log rank p=0.18
Multivariable Adjusted HR (95% CI):
Asian vs. White 0.78 (0.19–3.14)
Black vs. White 1.71 (1.01–2.89)
Hispanic vs. White 1.74 (0.95–3.18)
No. at risk:
Asian 57 19 <11 0 | 47 24 <11 <11 | 35 18 <11 0
Black 19 <11 <11 <11 | 495 216 77 <11 | 92 39 12 0
Hispanic 290 133 32 <11 | 81 31 <11 <11 | 102 41 17 <11
White 3864 2131 764 76 | 8775 4874 1928 300 | 7977 4598 1801 289

eTable 1. Characteristics of patients with uterine cancer according to SEER registry, 2000-2019											
**Characteristic**	**Hawaii**	**California**	**New Jersey**	**Georgia**	**New Mexico**	**Seattle (Puget Sound), Washington**	**Louisiana**	**Connecticut**	**Utah**	**Kentucky**	**Iowa**
	**(n=2,454)**	**(n=69,557)**	**(n=22,993)**	**(n=16,015)**	**(n=3,512)**	**(n=9,784)**	**(n=6,843)**	**(n=9,508)**	**(n=4,230)**	**(n=9,398)**	**(n=8,206)**
**Race**											
Asian	1,575 (64.2)	8,484 (12.2)	931 (4.1)	270 (1.7)	38 (1.1)	597 (6.1)	67 (1.0)	125 (1.3)	57 (1.4)	47 (0.5)	35 (0.4)
Black	15 (0.6)	3,912 (5.6)	2,197 (9.6)	4,326 (27.0)	51 (1.5)	256 (2.6)	1,961 (28.7)	683 (7.2)	19 (0.5)	495 (5.3)	92 (1.1)
Hispanic	150 (6.1)	15,149 (21.8)	2,024 (8.8)	571 (3.6)	1,248 (35.5)	349 (3.6)	146 (2.1)	689 (7.3)	290 (6.9)	81 (0.9)	102 (1.2)
White	714 (29.1)	42,012 (60.4)	17,841 (77.6)	10,848 (67.7)	2,175 (61.9)	8,582 (87.7)	4,669 (68.2)	8,011 (84.3)	3,864 (91.4)	8,775 (93.4)	7,977 (97.2)
**Age, years**											
<50	438 (17.9)	9,918 (14.3)	2,354 (10.2)	2,353 (14.7)	494 (14.1)	1,162 (11.9)	1,049 (15.3)	1,017 (10.7)	639 (15.1)	1,652 (17.6)	876 (10.7)
50-69	1,506 (61.4)	43,058 (61.9)	14,578 (63.4)	10,188 (63.6)	2,196 (62.5)	6,318 (64.6)	4,217 (61.6)	5,941 (62.5)	2,636 (62.3)	5,801 (61.7)	5,080 (61.9)
≥70	510 (20.8)	16,581 (23.8)	6,061 (26.4)	3,474 (21.7)	822 (23.4)	2,304 (23.6)	1,577 (23.1)	2,550 (26.8)	955 (22.6)	1,945 (20.7)	2,250 (27.4)
**Age, median (IQR)**	60 (52-68)	61 (54-69)	62 (56-70)	61 (54-68)	61 (54-69)	61 (55-69)	61 (54-69)	62 (55-70)	61 (54-69)	60 (53-68)	63 (56-70)
**Area-level annual income, $**											
<75,000	338 (13.8)	30,033 (43.2)	3,856 (16.8)	10,394 (64.9)	3,393 (96.6)	1,747 (17.9)	6,298 (92.0)	100 (1.1)	979 (23.1)	8,712 (92.7)	6,676 (81.4)
≥75,000	2,116 (86.2)	39,524 (56.8)	19,136 (83.2)	5,621 (35.1)	112 (3.2)	8,037 (82.1)	545 (8.0)	9,408 (99.0)	3,251 (76.9)	686 (7.3)	1,530 (18.6)
Unknown	0 (0.0)	0 (0.0)	<11	0 (0.0)	<11	0 (0.0)	0 (0.0)	0 (0.0)	0 (0.0)	0 (0.0)	0 (0.0)
**Marital status**											
Unmarried	1,027 (41.9)	31,020 (44.6)	9,528 (41.4)	7,200 (45.0)	1,439 (41.0)	3,972 (40.6)	3,254 (47.6)	4,244 (44.6)	1,587 (37.5)	3,771 (40.1)	3,147 (38.4)
Married	1,348 (54.9)	36,001 (51.8)	12,024 (52.3)	8,142 (50.8)	1,751 (49.9)	5,311 (54.3)	3,300 (48.2)	4,949 (52.1)	2,577 (60.9)	5,058 (53.8)	4,929 (60.1)
Unknown	79 (3.2)	2,536 (3.7)	1,441 (6.3)	673 (4.2)	322 (9.2)	501 (5.1)	289 (4.2)	315 (3.3)	66 (1.6)	569 (6.1)	130 (1.6)

© 2025 Meade CE et al. *JAMA Network Open*.

eTable 1. Characteristics of patients with uterine cancer according to SEER registry, 2000-2019											
**Characteristic**	**Hawaii**	**California**	**New Jersey**	**Georgia**	**New Mexico**	**Seattle (Puget Sound), Washington**	**Louisiana**	**Connecticut**	**Utah**	**Kentucky**	**Iowa**
**Rural/urban location**											
Urban	1,942 (79.1)	67,680 (97.3)	22,992 (100.0)	12,734 (79.5)	2,499 (71.2)	8,829 (90.2)	5,432 (79.4)	8,848 (93.1)	3,741 (88.4)	5,040 (53.6)	4,332 (52.8)
Rural	512 (20.9)	1,877 (2.7)	0 (0.0)	3,281 (20.5)	1,006 (28.6)	955 (9.8)	1,411 (20.6)	660 (6.9)	489 (11.6)	4,358 (46.4)	3,874 (47.2)
Unknown	0 (0.0)	0 (0.0)	<11	0 (0.0)	<11	0 (0.0)	0 (0.0)	0 (0.0)	0 (0.0)	0 (0.0)	0 (0.0)
**Year of diagnosis**											
2000-2004	459 (18.7)	12,244 (17.6)	4,375 (19.0)	2,409 (15.0)	569 (16.2)	1,613 (16.5)	1,341 (19.6)	1,971 (20.7)	616 (14.6)	1,708 (18.2)	1,646 (20.1)
2005-2009	527 (21.5)	14,842 (21.3)	5,366 (23.3)	3,188 (19.9)	748 (21.3)	2,237 (22.9)	1,416 (20.7)	2,228 (23.4)	936 (22.1)	2,054 (21.9)	1,874 (22.8)
2010-2014	693 (28.2)	19,808 (28.5)	6,369 (27.7)	4,594 (28.7)	1,061 (30.2)	2,877 (29.4)	1,913 (28.0)	2,557 (26.9)	1,268 (30.0)	2,649 (28.2)	2,295 (28.0)
2015-2019	775 (31.6)	22,663 (32.6)	6,883 (29.9)	5,824 (36.4)	1,134 (32.3)	3,057 (31.2)	2,173 (31.8)	2,752 (28.9)	1,410 (33.3)	2,987 (31.8)	2,391 (29.1)
**Histology**											
Low-grade endometrioid	1,578 (64.3)	41,517 (59.7)	14,413 (62.7)	8,325 (52.0)	2,090 (59.5)	3,608 (36.9)	4,027 (58.9)	5,760 (60.6)	2,648 (62.6)	6,087 (64.8)	5,706 (69.5)
High-grade endometrioid	277 (11.3)	6,922 (10.0)	2,499 (10.9)	1,574 (9.8)	342 (9.7)	914 (9.3)	824 (12.0)	838 (8.8)	347 (8.2)	1,066 (11.3)	777 (9.5)
Unknown-grade endometrioid	203 (8.3)	7,569 (10.9)	1,978 (8.6)	2,665 (16.6)	473 (13.5)	3,718 (38.0)	581 (8.5)	884 (9.3)	631 (14.9)	698 (7.4)	327 (4.0)
Serous	121 (4.9)	4,602 (6.6)	1,515 (6.6)	1,311 (8.2)	223 (6.4)	587 (6.0)	544 (8.0)	642 (6.8)	188 (4.4)	486 (5.2)	439 (5.4)
Carcinosarcoma	67 (2.7)	2,274 (3.3)	832 (3.6)	793 (5.0)	125 (3.6)	281 (2.9)	297 (4.3)	354 (3.7)	117 (2.8)	247 (2.6)	205 (2.5)
Clear cell	50 (2.0)	955 (1.4)	265 (1.2)	195 (1.2)	42 (1.2)	95 (1.0)	86 (1.3)	112 (1.2)	34 (0.8)	74 (0.8)	86 (1.1)
Mixed epithelial	103 (4.2)	4,037 (5.8)	973 (4.2)	702 (4.4)	126 (3.6)	387 (4.0)	287 (4.2)	725 (7.6)	170 (4.0)	577 (6.1)	534 (6.5)
Sarcoma	55 (2.2)	1,681 (2.4)	518 (2.3)	450 (2.8)	91 (2.6)	194 (2.0)	197 (2.9)	193 (2.0)	95 (2.3)	163 (1.7)	132 (1.6)
**Tumor stage**											

© 2025 Meade CE et al. *JAMA Network Open*.

eTable 1. Characteristics of patients with uterine cancer according to SEER registry, 2000-2019											
**Characteristic**	**Hawaii**	**California**	**New Jersey**	**Georgia**	**New Mexico**	**Seattle (Puget Sound), Washington**	**Louisiana**	**Connecticut**	**Utah**	**Kentucky**	**Iowa**
I	1,870 (76.2)	52,473 (75.4)	17,603 (76.6)	11,762 (73.4)	2,651 (75.5)	7,593 (77.6)	5,040 (73.7)	7,326 (77.1)	3,380 (79.9)	7,539 (80.2)	6,603 (80.5)
II	117 (4.8)	4,196 (6.0)	1,499 (6.5)	1,099 (6.9)	244 (7.0)	511 (5.2)	436 (6.4)	583 (6.1)	203 (4.8)	504 (5.4)	437 (5.3)
III	322 (13.1)	8,589 (12.4)	2,672 (11.6)	2,057 (12.8)	428 (12.2)	1,190 (12.2)	892 (13.0)	1,127 (11.9)	453 (10.7)	879 (9.4)	757 (9.2)
IV	145 (5.9)	4,299 (6.2)	1,219 (5.3)	1,097 (6.9)	189 (5.4)	490 (5.0)	475 (6.9)	472 (5.0)	194 (4.6)	476 (5.1)	409 (5.0)
**Surgery**											
Tumor destruction or excision	<11	431 (0.6)	210 (0.9)	215 (1.3)	62 (1.8)	35 (0.4)	57 (0.8)	80 (0.8)	32 (0.8)	56 (0.6)	26 (0.3)
Subtotal or supracervical hysterectomy	21 (0.9)	917 (1.3)	348 (1.5)	233 (1.5)	41 (1.2)	33 (0.3)	101 (1.5)	68 (0.7)	46 (1.1)	106 (1.1)	31 (0.4)
Total abdominal hysterectomy with or without bilateral salpingo-oophorectomy	2,354 (95.9)	64,351 (92.5)	21,449 (93.3)	14,384 (89.8)	3,163 (90.1)	9,381 (95.9)	6,367 (93.0)	8,841 (93.0)	3,620 (85.6)	8,718 (92.8)	7,956 (97.0)
Modified radical or extended hysterectomy	64 (2.6)	3,773 (5.4)	950 (4.1)	1,158 (7.2)	238 (6.8)	334 (3.4)	252 (3.7)	508 (5.3)	532 (12.6)	501 (5.3)	189 (2.3)
Surgery, not otherwise specified	<11	85 (0.1)	36 (0.2)	25 (0.2)	<11	<11	66 (1.0)	11 (0.1)	0 (0.0)	17 (0.2)	<11
**Chemotherapy**											
No/Unknown	1,888 (76.9)	57,385 (82.5)	18,753 (81.6)	12,502 (78.1)	2,891 (82.3)	7,837 (80.1)	5,527 (80.8)	7,393 (77.8)	3,710 (87.7)	7,996 (85.1)	6,972 (85.0)
Yes	566 (23.1)	12,172 (17.5)	4,240 (18.4)	3,513 (21.9)	621 (17.7)	1,947 (19.9)	1,316 (19.2)	2,115 (22.2)	520 (12.3)	1,402 (14.9)	1,234 (15.0)
**Radiation**											

© 2025 Meade CE et al. *JAMA Network Open*.

eTable 1. Characteristics of patients with uterine cancer according to SEER registry, 2000-2019

Characteristic	Hawaii	California	New Jersey	Georgia	New Mexico	Seattle (Puget Sound), Washington	Louisiana	Connecticut	Utah	Kentucky	Iowa
None	2,053 (83.7)	52,678 (75.7)	15,535 (67.6)	11,118 (69.4)	2,632 (74.9)	6,810 (69.6)	4,613 (67.4)	5,516 (58.0)	3,106 (73.4)	7,190 (76.5)	5,896 (71.9)
External beam radiation therapy	174 (7.1)	7,506 (10.8)	2,275 (9.9)	1,733 (10.8)	322 (9.2)	910 (9.3)	982 (14.4)	730 (7.7)	409 (9.7)	714 (7.6)	812 (9.9)
Vaginal brachytherapy	87 (3.6)	5,422 (7.8)	3,397 (14.8)	1,911 (11.9)	280 (8.0)	1,485 (15.2)	797 (11.7)	2,634 (27.7)	500 (11.8)	704 (7.5)	932 (11.4)
External beam radiation therapy with vaginal brachytherapy	116 (4.7)	2,787 (4.0)	1,565 (6.8)	950 (5.9)	198 (5.6)	430 (4.4)	405 (5.9)	437 (4.6)	188 (4.4)	712 (7.6)	529 (6.5)
Unknown	24 (1.0)	1,164 (1.7)	221 (1.0)	303 (1.9)	80 (2.3)	149 (1.5)	46 (0.7)	191 (2.0)	27 (0.6)	78 (0.8)	37 (0.5)

© 2025 Meade CE et al. *JAMA Network Open*.

eFigure 2. Association between race and ethnicity and survival among Asian patients stratified by SEER registry, histology, and stage

eFigure 2 legend. Compared with White patients overall, Asian patients diagnosed with non-endometrioid or advanced-stage tumors had better cancer-specific survival. Within the California registry, Asian patients diagnosed with advanced-stage tumors had better cancer-specific survival compared with White patients.

Registry and Subgroup	deaths/N (%)	HR (95% CI)
**Overall**		
Low–grade endometrioid	288/7,157 (4.0)	0.96 (0.84–1.10)
High–grade endometrioid	270/1,254 (21.5)	0.92 (0.80–1.05)
Non–endometrioid	557/1,989 (28.0)	0.91 (0.83–1.00)
Sarcoma	88/332 (26.5)	0.88 (0.69–1.11)
Early stage (I)	372/9,140 (4.1)	0.90 (0.80–1.01)
Advanced stage (II/III/IV)	895/3,086 (29.0)	0.92 (0.86–0.99)
**Hawaii (DI=76.0%)**		
Low–grade endometrioid	39/1,033 (3.8)	0.67 (0.38–1.18)
High–grade endometrioid	46/157 (29.3)	1.70 (0.89–3.25)
Non–endometrioid	65/222 (29.3)	1.09 (0.65–1.81)
Sarcoma	11/40 (27.5)	0.84 (0.11–6.66)
Early stage (I)	50/1,199 (4.2)	0.77 (0.47–1.24)
Advanced stage (II/III/IV)	119/376 (31.7)	1.23 (0.85–1.78)
**California (DI=69.7%)**		
Low–grade endometrioid	207/4,970 (4.2)	0.97 (0.84–1.13)
High–grade endometrioid	190/887 (21.4)	0.87 (0.74–1.02)
Non–endometrioid	399/1,436 (27.8)	0.91 (0.82–1.01)
Sarcoma	62/218 (28.4)	0.95 (0.72–1.26)
Early stage (I)	264/6,305 (4.2)	0.91 (0.80–1.04)
Advanced stage (II/III/IV)	634/2,179 (29.1)	0.92 (0.85–1.00)
**New Jersey (DI=65.8%)**		
Low–grade endometrioid	21/566 (3.7)	1.14 (0.73–1.77)
High–grade endometrioid	17/107 (15.9)	1.03 (0.63–1.68)
Non–endometrioid	40/148 (27.0)	0.92 (0.66–1.26)
Sarcoma	<11	0.76 (0.30–1.93)
Early stage (I)	26/711 (3.7)	0.82 (0.55–1.22)
Advanced stage (II/III/IV)	59/220 (26.8)	0.89 (0.69–1.16)
**Georgia (DI=64.1%)**		
Low–grade endometrioid	<11	1.03 (0.46–2.31)
High–grade endometrioid	<11	0.34 (0.05–2.42)
Non–endometrioid	14/55 (25.5)	0.75 (0.44–1.27)
Sarcoma	<11	0.82 (0.24–2.84)
Early stage (I)	<11	0.87 (0.39–1.95)
Advanced stage (II/III/IV)	20/80 (25.0)	0.75 (0.48–1.17)
**New Mexico (DI=63.0%)**		
Low–grade endometrioid	<11	0.69 (0.09–5.52)
High–grade endometrioid	<11	3.69 (0.48–28.49)
Non–endometrioid	<11	0.48 (0.06–3.59)
Sarcoma	0/<11 (0.0)	–
Early stage (I)	0/23 (0.0)	–
Advanced stage (II/III/IV)	<11	0.77 (0.24–2.45)
**Seattle (DI=60.1%)**		
Low–grade endometrioid	<11	1.23 (0.56–2.72)
High–grade endometrioid	<11	0.63 (0.31–1.28)
Non–endometrioid	25/70 (35.7)	0.95 (0.62–1.44)
Sarcoma	<11	0.60 (0.20–1.78)
Early stage (I)	17/456 (3.7)	1.00 (0.61–1.64)
Advanced stage (II/III/IV)	39/141 (27.7)	0.93 (0.67–1.30)
**Louisiana (DI=58.6%)**		
Low–grade endometrioid	<11	0.76 (0.11–5.49)
High–grade endometrioid	<11	0.75 (0.10–5.50)
Non–endometrioid	<11	1.28 (0.31–5.26)
Sarcoma	<11	2.11 (0.26–17.23)
Early stage (I)	<11	1.20 (0.29–4.88)
Advanced stage (II/III/IV)	<11	0.69 (0.22–2.15)
**Connecticut (DI=55.7%)**		
Low–grade endometrioid	<11	1.74 (0.55–5.51)
High–grade endometrioid	<11	3.75 (0.90–15.63)
Non–endometrioid	<11	1.12 (0.46–2.74)
Sarcoma	<11	1.13 (0.15–8.66)
Early stage (I)	<11	1.61 (0.66–3.95)
Advanced stage (II/III/IV)	<11	0.97 (0.46–2.07)
**Utah (DI=40.7%)**		
Low–grade endometrioid	<11	2.62 (0.64–10.74)
High–grade endometrioid	<11	1.02 (0.23–4.58)
Non–endometrioid	<11	1.63 (0.21–12.49)
Sarcoma	<11	0.17 (0.02–1.87)
Early stage (I)	<11	0.92 (0.22–3.78)
Advanced stage (II/III/IV)	<11	2.78 (0.98–7.88)
**Kentucky (DI=32.8%)**		
Low–grade endometrioid	<11	1.40 (0.19–10.11)
High–grade endometrioid	<11	0.64 (0.09–4.60)
Non–endometrioid	<11	1.17 (0.37–3.69)
Sarcoma	0/<11 (0.0)	–
Early stage (I)	0/35 (0.0)	–
Advanced stage (II/III/IV)	<11	1.24 (0.51–3.01)
**Iowa (DI=30.8%)**		
Low–grade endometrioid	0/22 (0.0)	–
High–grade endometrioid	0/<11 (0.0)	–
Non–endometrioid	<11	1.96 (0.48–7.98)
Sarcoma	0/<11 (0.0)	–
Early stage (I)	0/26 (0.0)	–
Advanced stage (II/III/IV)	<11	0.95 (0.24–3.85)

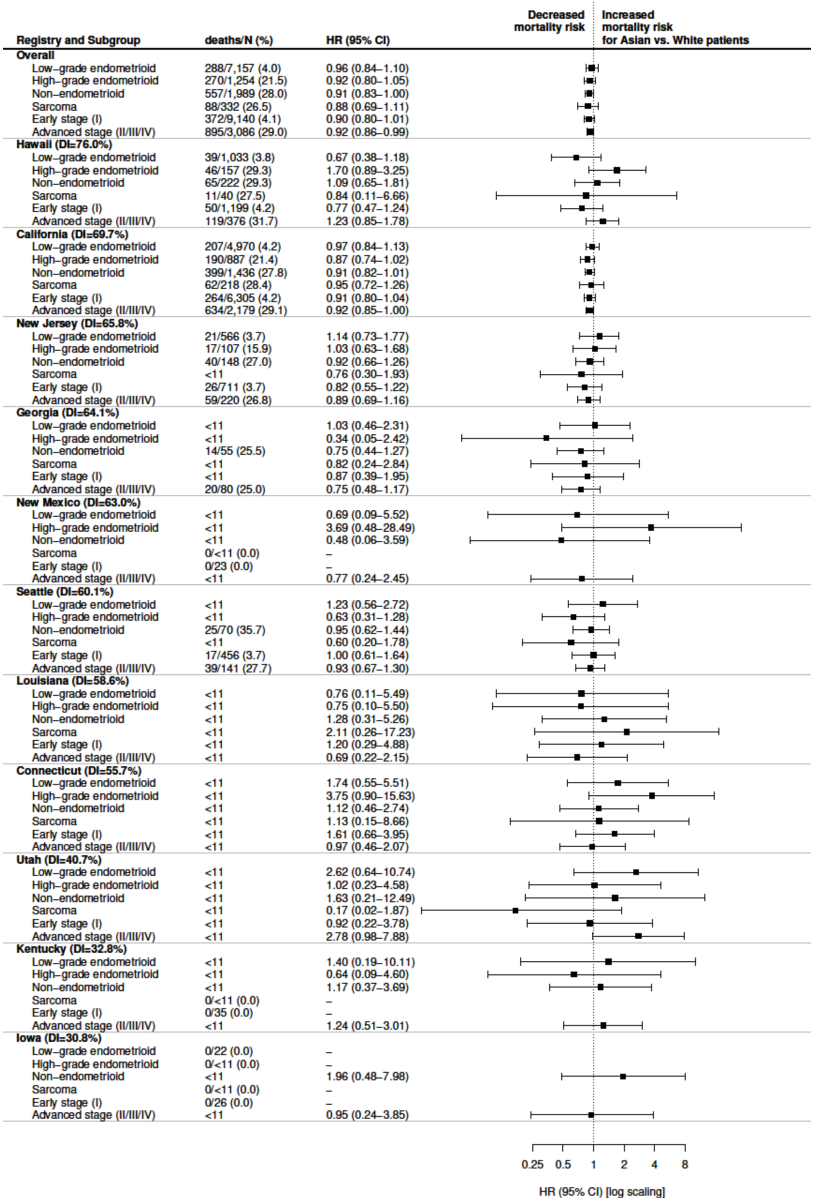

© 2025 Meade CE et al. *JAMA Network Open*.

eFigure 3. Association between race and ethnicity and survival among Black patients stratified by SEER registry, histology, and stage

eFigure 3 legend. Compared with White patients overall, Black patients had worse cancer-specific survival in all histology and stage subgroups. Worse cancer-specific survival was also noted for Black patients in one or more tumor-defined subgroups within Georgia, California, New Jersey, Connecticut, Louisiana, Seattle, and Iowa.

© 2025 Meade CE et al. *JAMA Network Open*.

Registry and Subgroup	deaths/N (%)	HR (95% CI)
**Overall**		
Low–grade endometrioid	358/5,540 (6.5)	1.50 (1.33–1.68)
High–grade endometrioid	512/1,691 (30.3)	1.30 (1.17–1.44)
Non–endometrioid	1,827/4,712 (38.8)	1.42 (1.33–1.50)
Sarcoma	265/708 (37.4)	1.39 (1.18–1.63)
Early stage (I)	890/9,123 (9.8)	1.47 (1.35–1.59)
Advanced stage (II/III/IV)	2,192/4,884 (44.9)	1.25 (1.18–1.32)
**Hawaii (DI=76.0%)**		
Low–grade endometrioid	0/<11 (0.0)	–
High–grade endometrioid	0/<11 (0.0)	–
Non–endometrioid	<11	0.72 (0.09–5.44)
Sarcoma	<11	2.47 (0.08–73.99)
Early stage (I)	<11	0.87 (0.11–6.97)
Advanced stage (II/III/IV)	<11	0.61 (0.08–4.65)
**California (DI=69.7%)**		
Low–grade endometrioid	98/1,587 (6.2)	1.42 (1.16–1.75)
High–grade endometrioid	144/451 (31.9)	1.19 (0.99–1.41)
Non–endometrioid	566/1,429 (39.6)	1.45 (1.31–1.59)
Sarcoma	79/172 (45.9)	1.58 (1.22–2.05)
Early stage (I)	270/2,582 (10.5)	1.39 (1.21–1.58)
Advanced stage (II/III/IV)	643/1,330 (48.4)	1.30 (1.19–1.42)
**New Jersey (DI=65.8%)**		
Low–grade endometrioid	57/913 (6.2)	1.28 (0.97–1.71)
High–grade endometrioid	92/283 (32.5)	1.45 (1.14–1.83)
Non–endometrioid	285/710 (40.1)	1.44 (1.24–1.67)
Sarcoma	28/102 (27.5)	0.96 (0.60–1.52)
Early stage (I)	132/1,420 (9.3)	1.41 (1.16–1.71)
Advanced stage (II/III/IV)	346/777 (44.5)	1.26 (1.11–1.44)
**Georgia (DI=64.1%)**		
Low–grade endometrioid	104/1,550 (6.7)	1.49 (1.18–1.89)
High–grade endometrioid	147/490 (30.0)	1.34 (1.08–1.67)
Non–endometrioid	566/1,434 (39.5)	1.43 (1.25–1.63)
Sarcoma	88/244 (36.1)	1.59 (1.08–2.33)
Early stage (I)	273/2,762 (9.9)	1.69 (1.42–2.01)
Advanced stage (II/III/IV)	685/1,564 (43.8)	1.23 (1.10–1.38)
**New Mexico (DI=63.0%)**		
Low–grade endometrioid	<11	1.86 (0.43–8.05)
High–grade endometrioid	<11	1.91 (0.43–8.43)
Non–endometrioid	<11	1.08 (0.47–2.49)
Sarcoma	0/<11 (0.0)	–
Early stage (I)	<11	0.55 (0.07–4.29)
Advanced stage (II/III/IV)	11/22 (50.0)	1.37 (0.71–2.64)
**Seattle (DI=60.1%)**		
Low–grade endometrioid	<11	2.27 (0.70–7.37)
High–grade endometrioid	<11	2.23 (1.03–4.83)
Non–endometrioid	29/81 (35.8)	1.03 (0.70–1.52)
Sarcoma	<11	0.76 (0.23–2.59)
Early stage (I)	<11	0.54 (0.25–1.17)
Advanced stage (II/III/IV)	39/85 (45.9)	1.20 (0.86–1.67)
**Louisiana (DI=58.6%)**		
Low–grade endometrioid	69/846 (8.2)	2.08 (1.54–2.82)
High–grade endometrioid	79/287 (27.5)	1.18 (0.88–1.58)
Non–endometrioid	218/590 (37.0)	1.29 (1.04–1.60)
Sarcoma	41/104 (39.4)	1.40 (0.81–2.42)
Early stage (I)	129/1,257 (10.3)	1.77 (1.38–2.28)
Advanced stage (II/III/IV)	287/704 (40.8)	1.06 (0.89–1.26)
**Connecticut (DI=55.7%)**		
Low–grade endometrioid	13/274 (4.7)	1.82 (1.02–3.23)
High–grade endometrioid	18/71 (25.4)	1.92 (1.13–3.27)
Non–endometrioid	93/266 (35.0)	1.47 (1.16–1.87)
Sarcoma	13/32 (40.6)	1.33 (0.62–2.84)
Early stage (I)	41/445 (9.2)	1.97 (1.39–2.80)
Advanced stage (II/III/IV)	104/238 (43.7)	1.40 (1.12–1.76)
**Utah (DI=40.7%)**		
Low–grade endometrioid	0/<11 (0.0)	–
High–grade endometrioid	0/<11 (0.0)	–
Non–endometrioid	<11	2.82 (0.87–9.12)
Sarcoma	0/0 (0.0)	–
Early stage (I)	<11	1.04 (0.14–7.58)
Advanced stage (II/III/IV)	<11	1.48 (0.36–6.10)
**Kentucky (DI=32.8%)**		
Low–grade endometrioid	<11	1.26 (0.66–2.40)
High–grade endometrioid	21/69 (30.4)	1.40 (0.88–2.25)
Non–endometrioid	50/149 (33.6)	1.18 (0.86–1.61)
Sarcoma	11/26 (42.3)	2.07 (0.85–5.05)
Early stage (I)	30/360 (8.3)	1.20 (0.81–1.78)
Advanced stage (II/III/IV)	64/135 (47.4)	1.13 (0.86–1.49)
**Iowa (DI=30.8%)**		
Low–grade endometrioid	<11	1.48 (0.36–6.03)
High–grade endometrioid	<11	1.35 (0.32–5.68)
Non–endometrioid	<11	2.01 (1.00–4.01)
Sarcoma	<11	0.83 (0.10–6.90)
Early stage (I)	<11	1.53 (0.62–3.78)
Advanced stage (II/III/IV)	<11	1.51 (0.79–2.90)

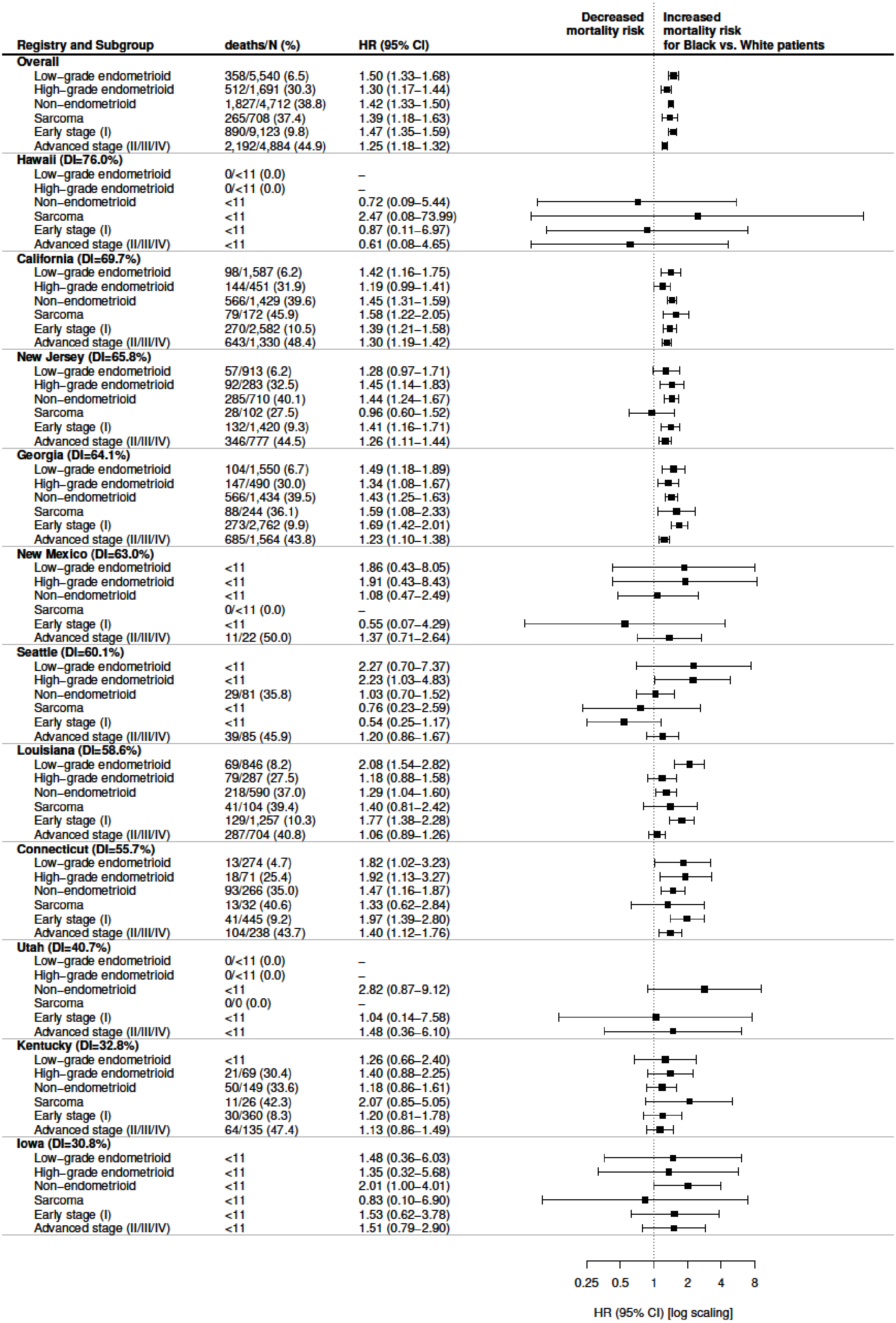

eFigure 4. Association between race and ethnicity and survival among Hispanic patients stratified by SEER registry, histology, and stage

eFigure 4 legend. Compared with White patients, no significant differences were noted among Hispanic patients in the overall sample. In location stratified models, worse cancer-specific survival was noted for Hispanic patients in one or more tumor-defined subgroups within Hawaii, Georgia, Louisiana, Kentucky, and Iowa.

© 2025 Meade CE et al. *JAMA Network Open*.

Registry and Subgroup	deaths/N (%)	HR (95% CI)
**Overall**		
Low–grade endometrioid	471/12,312 (3.8)	1.01 (0.91–1.11)
High–grade endometrioid	391/1,889 (20.7)	0.92 (0.83–1.03)
Non–endometrioid	1,001/3,386 (29.6)	1.04 (0.96–1.11)
Sarcoma	227/768 (29.6)	1.15 (0.98–1.36)
Early stage (I)	689/15,708 (4.4)	1.03 (0.95–1.12)
Advanced stage (II/III/IV)	1,538/5,091 (30.2)	1.00 (0.94–1.05)
**Hawaii (DI=76.0%)**		
Low–grade endometrioid	<11	1.87 (0.74–4.71)
High–grade endometrioid	<11	1.76 (0.57–5.43)
Non–endometrioid	<11	3.99 (1.72–9.25)
Sarcoma	<11	0.09 (0.00–8.76)
Early stage (I)	<11	1.24 (0.55–2.76)
Advanced stage (II/III/IV)	14/28 (50.0)	3.28 (1.75–6.13)
**California (DI=69.7%)**		
Low–grade endometrioid	339/9,030 (3.8)	0.96 (0.85–1.09)
High–grade endometrioid	284/1,372 (20.7)	0.92 (0.80–1.05)
Non–endometrioid	739/2,501 (29.6)	1.02 (0.93–1.11)
Sarcoma	171/542 (31.6)	1.15 (0.94–1.40)
Early stage (I)	510/11,406 (4.5)	1.01 (0.91–1.12)
Advanced stage (II/III/IV)	1,113/3,743 (29.7)	0.98 (0.91–1.05)
**New Jersey (DI=65.8%)**		
Low–grade endometrioid	46/1,228 (3.8)	0.99 (0.73–1.34)
High–grade endometrioid	44/200 (22.0)	1.06 (0.77–1.46)
Non–endometrioid	94/323 (29.1)	0.94 (0.75–1.17)
Sarcoma	22/86 (25.6)	1.05 (0.64–1.72)
Early stage (I)	57/1,534 (3.7)	0.81 (0.61–1.06)
Advanced stage (II/III/IV)	159/490 (32.5)	1.02 (0.86–1.20)
**Georgia (DI=64.1%)**		
Low–grade endometrioid	18/294 (6.1)	1.60 (0.98–2.62)
High–grade endometrioid	13/43 (30.2)	1.34 (0.76–2.37)
Non–endometrioid	32/96 (33.3)	1.41 (0.98–2.02)
Sarcoma	<11	1.63 (0.67–4.02)
Early stage (I)	22/413 (5.3)	1.63 (1.06–2.53)
Advanced stage (II/III/IV)	52/158 (32.9)	1.16 (0.87–1.54)
**New Mexico (DI=63.0%)**		
Low–grade endometrioid	31/724 (4.3)	1.05 (0.67–1.64)
High–grade endometrioid	22/127 (17.3)	0.58 (0.34–1.01)
Non–endometrioid	51/191 (26.7)	0.96 (0.67–1.37)
Sarcoma	<11	0.70 (0.26–1.94)
Early stage (I)	43/961 (4.5)	1.32 (0.88–1.96)
Advanced stage (II/III/IV)	82/287 (28.6)	0.91 (0.69–1.19)
**Seattle (DI=60.1%)**		
Low–grade endometrioid	<11	0.91 (0.28–2.90)
High–grade endometrioid	<11	0.63 (0.19–2.07)
Non–endometrioid	20/54 (37.0)	1.39 (0.87–2.20)
Sarcoma	<11	0.25 (0.03–2.06)
Early stage (I)	<11	0.89 (0.44–1.80)
Advanced stage (II/III/IV)	29/82 (35.4)	1.34 (0.92–1.96)
**Louisiana (DI=58.6%)**		
Low–grade endometrioid	<11	1.20 (0.44–3.28)
High–grade endometrioid	<11	1.02 (0.41–2.55)
Non–endometrioid	<11	0.99 (0.46–2.14)
Sarcoma	<11	4.52 (1.32–15.44)
Early stage (I)	<11	1.77 (0.82–3.81)
Advanced stage (II/III/IV)	14/36 (38.9)	1.11 (0.65–1.91)
**Connecticut (DI=55.7%)**		
Low–grade endometrioid	13/439 (3.0)	1.11 (0.63–1.98)
High–grade endometrioid	<11	1.07 (0.50–2.28)
Non–endometrioid	28/113 (24.8)	1.18 (0.79–1.74)
Sarcoma	<11	0.72 (0.27–1.97)
Early stage (I)	16/520 (3.1)	0.95 (0.57–1.59)
Advanced stage (II/III/IV)	38/169 (22.5)	0.93 (0.66–1.30)
**Utah (DI=40.7%)**		
Low–grade endometrioid	<11	1.87 (0.93–3.75)
High–grade endometrioid	<11	0.57 (0.17–1.85)
Non–endometrioid	14/46 (30.4)	0.74 (0.42–1.33)
Sarcoma	<11	1.88 (0.50–7.04)
Early stage (I)	<11	1.03 (0.52–2.05)
Advanced stage (II/III/IV)	25/64 (39.1)	0.97 (0.63–1.48)
**Kentucky (DI=32.8%)**		
Low–grade endometrioid	0/49 (0.0)	–
High–grade endometrioid	<11	0.62 (0.15–2.61)
Non–endometrioid	<11	3.82 (1.53–9.57)
Sarcoma	<11	3.70 (0.38–35.76)
Early stage (I)	<11	1.71 (0.63–4.61)
Advanced stage (II/III/IV)	<11	1.45 (0.64–3.30)
**Iowa (DI=30.8%)**		
Low–grade endometrioid	<11	0.75 (0.18–3.06)
High–grade endometrioid	<11	5.53 (1.27–24.03)
Non–endometrioid	<11	0.68 (0.17–2.77)
Sarcoma	<11	4.72 (1.30–17.08)
Early stage (I)	<11	2.13 (0.85–5.32)
Advanced stage (II/III/IV)	<11	1.11 (0.49–2.51)

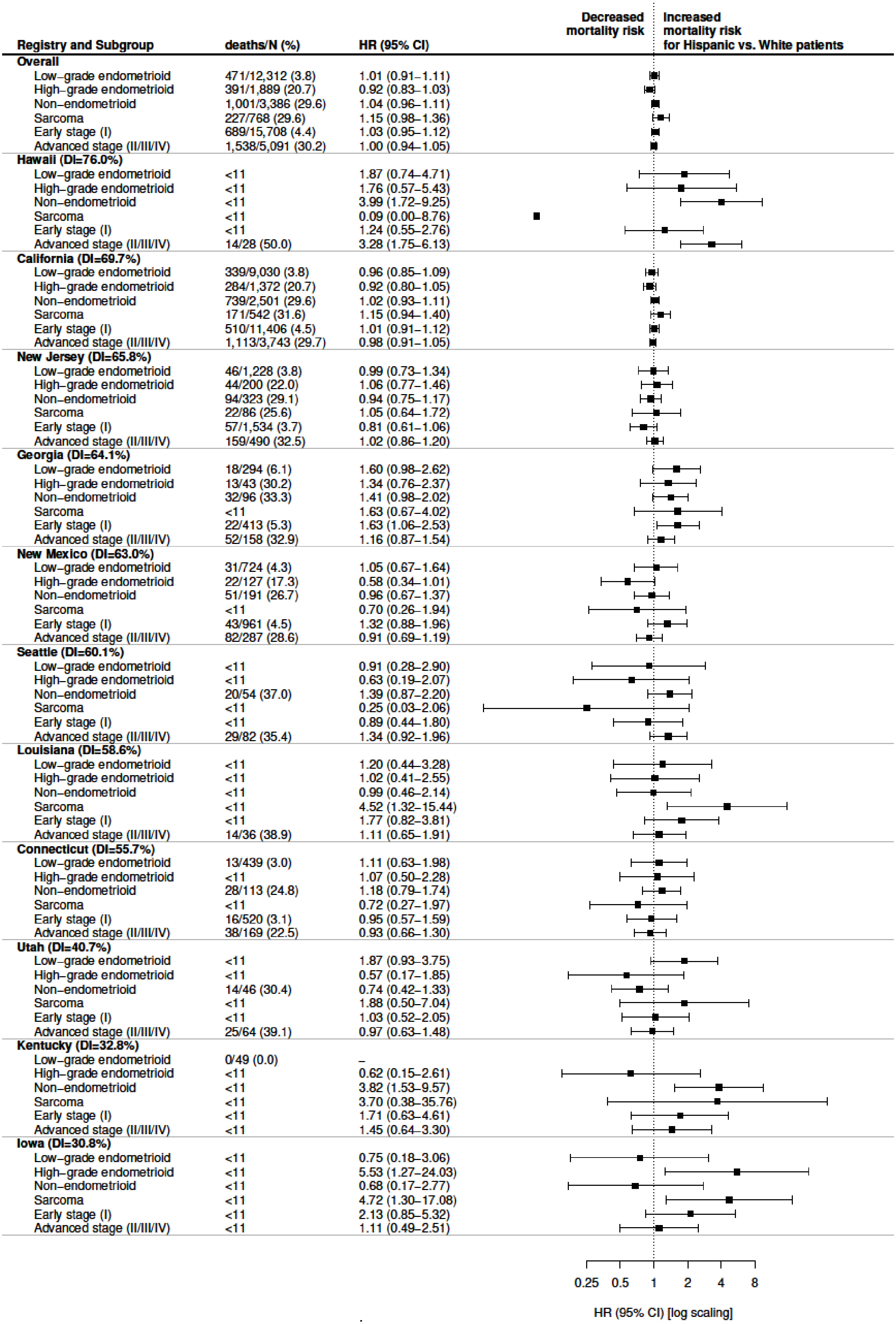

.
