# Supplementary material for: Geographic Variation of Racial and Ethnic Differences in Uterine Cancer Survival
Source: JAMA Netw Open. 2025 Apr 25;8(4):e257227. doi: 10.1001/jamanetworkopen.2025.7227 (PMC12032568; doi:10.1001/jamanetworkopen.2025.7227)
Supplement: Supplement 2. — Data Sharing Statement [file jamanetwopen-e257227-s002.pdf]

Data Sharing Statement

Meade. Geographic Variation of Racial and Ethnic Differences in Uterine Cancer Survival. *JAMA Netw Open*. Published April 25, 2025. doi:10.1001/jamanetworkopen.2025.7227

Data
Data available: No

Additional Information
Explanation for why data not available: The data for this study were acquired by application to the restricted access Surveillance, Epidemiology, End Results Program and the data use agreement does not allow data sharing.
